# The Sick and Wounded

**Published:** 1920-12-25

**Authors:** 


**THE PUBLIC WELL-BEING**—(*continued*).

### The Sick and Wounded.

### A CONSULTING SURGEON'S MEMORIES OF THE NEAR EAST.

COLONEL A. H. TUBBY, who was a consulting surgeon to the Mediterranean and Egyptian Expeditionary Forces from 1915 to 1919, has written an interesting (and modest) book of his personal experiences in Gallipoli and Egypt. He is one of the civilian hospital surgeons who were selected by the War Office to serve abroad nominally in a consultant capacity—though the duties of these consultants on active service became so varied as often to defy classification. The services of such eminent men as these, dealing with urgent and complex problems having new and unexpected aspects in different theatres of war, were not only extremely valuable in the emergencies of the campaign; but it is reasonable to hope that the experiments which they introduced and the lessons which they learnt in an unexampled opportunity will be applied with great advantage for the welfare of the civilian community in the future. A public health official told the present writer a short time ago that he was rather disappointed by the comparative poverty of new and valuable ideas brought back from their war experiences by the medical profession, and he more than hinted that the service of the public health had not gained as much as it was entitled to expect from nearly five years' contact with the realities of war and the mass problems which were then forced upon the attention of the best minds. The writer replied by advising this critic to study attentively the discussions and summaries published periodically in the "International Journal of Public Health," produced monthly by the League of Red Cross Societies, and suggested that if he went no further he would have to revise his opinion considerably. But in justice to the medical profession, it must be borne in mind that war surgery and war hygiene deal with conditions which never arise in peacetime in civilised communities. Surgery has learnt little from the war that is useful for civil practice, though much that will be invaluable should a great war occur in the future.

Colonel Tubby, in his book, gives us a fascinating record of his work, of the stupendous difficulties that had to be faced in the most anxious campaign of the whole war, and of the heroic and resourceful manner in which they were surmounted or mitigated. In 1915 the work accomplished in Alexandria, a by no means favourable environment for hospitals on a large scale, was extremely arduous. Colonel Tubby shows how

> rapidly and effectively the G.O.C. Force in Egypt, the D.M.S. Egypt, and their subordinates, had risen to the emergency and provided, within the space of two months, several times more than the number of beds which had been thought necessary when the campaign was planned. The lavish and unstinted help rendered by all sections of the community, particularly by ladies, should not be passed over without reference, and an expression of our profound gratitude must not be omitted.

The early days of May 1915 were the times of greatest strain on the hospitals.

> At one of them patients were arriving from Gallipoli before even the beds were put up, and the medical and nursing staffs were then working as orderlies. There was a shortage of sisters and nurses; we know of one matron who did her own duties during the day and acted as sister-in-charge of the operating-theatre at night. This meant continuous work for several days and nights in succession, and when it is recorded that a "khamseen," or a sand-laden south wind, was blowing from the desert at the time, and the temperature in the bell-tents rose to 109° F. during the day, it is possible to realise faintly what a tax upon health and strength such labours involved.
>
> On one occasion in a hospital, when a convoy of 600 arrived from Gallipoli, there were only three nursing sisters available for duty, which was most exacting, as many of the patients were grievously

**THE PUBLIC WELL-BEING—(*continued*).**

hurt, and most of them septic. Some assistance was obtained from Government dispensary nurses, and the civilian ladies of Alexandria volunteered their help at once in the wards. Civilian men helped in various ways, acting as clerks and orderlies; thus the crisis was tided over.

During the summer of that year the wards were infested with flies, and the sufferings of the wounded were aggravated by these pests, which the authorities had not then learned how to deal with, and which spread infection from typhoid, paratyphoid, and dysenteric cases.

The author appears to have had ample opportunities of seeing the work of the late Surgeon-General William Babtie, V.C. This officer has been much criticised, not alone by irresponsible journalists, but also by those who saw his work at first hand. It is only fair to record Col. Tubby's contrary opinion of him.

It appeared (writes the author) that he had been ordered to the Mediterranean from India to co-ordinate the arrangements at Gallipoli, Mudros, in Egypt, and on the lines of communication. It was a truly herculean task which had been imposed upon him, and one which might have depressed a less plucky and resilient nature. In him there could be noticed a strain of Celtic quickness and intensity, combined with ready sympathy, and more than a touch of humour. He was a man who could be relied upon to secure the best work of which they were capable from his subordinates.

Speaking personally, his administrative powers, his grasp of the problems submitted to him, his accessibility, his readiness to listen to suggestions, and, if approved, to act upon them, all compelled my admiration. I am clearly of opinion that the criticisms which were made upon his administration were uncalled for, and often ungenerous. The basic causes underlying any deficiencies in the medical and surgical work during the Gallipoli campaign must be sought elsewhere than in the Army Medical Service and its representatives.

Having thus aroused our curiosity, Colonel Tubby, perhaps with wise restraint, does not proceed to satisfy it as to where "the basic causes" were actually to be found.

With regard to the causes of our failure at Gallipoli, he places primarily the prevalence of diarrhœa and dysentery, painting a picture of the conditions under which our men lived that is the more terrible because it is simply drawn without any striving after effect; secondarily, the enervating effects of the climate on men fresh from home. "Nothing else," he adds, "would have held in check the determination of our troops to break through the Turkish defences. Widely prevalent disease and bad climatic conditions kept our men too low for so great a task."

In an extremely interesting chapter on the sick and convalescent soldier Colonel Tubby, while paying a well-deserved tribute to the eagerness of the average soldier to return to duty, gives examples of the methods of malingering employed in a minority of cases, and proceeds:

The general conclusions of this matter of unwillingness and malingering is that all, or nearly all, of us are nervous of ourselves in warfare. Some can control their apprehensiveness and put aside all deterrents to active service; others have only partial control, and are swayed by any kind of excuse; and a third class have so nearly lost self-control that they lend themselves, consciously or unconsciously, to trickery and deceit.

This book, of special interest though of course it is to the medical profession, may also be read with profit and interest by a much wider public, and not least of all by civilian arm-chair critics of our war-time medical organisations.

[*A Consulting Surgeon in the East.* By A. H. Tubby, C.B., C.M.G., M.S. (Lond.), F.R.C.S. (Eng.). Price 15s. net. (London: Christophers, 22 Berners Street, W. 1.)]

